# Pyruvate dehydrogenase kinases (PDKs): an overview toward clinical applications

**DOI:** 10.1042/BSR20204402

**Published:** 2021-04-07

**Authors:** Xiuxiu Wang, Xiaoyue Shen, Yuting Yan, Hongmin Li

**Affiliations:** 1 Provincial Key Laboratory of Biotechnology of Shaanxi, College of Life Sciences, Northwest University, Taibai North Rd 229, Xi’an, Shaanxi Province 710069, China; 2 Key Laboratory of Resource Biology and Biotechnology in Western China, Ministry of China, Taibai North Rd 229, Xi'an, Shaanxi Province 710069, China

**Keywords:** pyruvate dehydrogenase complex, pyruvate dehydrogenase kinase, pyruvate

## Abstract

Pyruvate dehydrogenase kinase (PDK) can regulate the catalytic activity of pyruvate decarboxylation oxidation via the mitochondrial pyruvate dehydrogenase complex, and it further links glycolysis with the tricarboxylic acid cycle and ATP generation. This review seeks to elucidate the regulation of PDK activity in different species, mainly mammals, and the role of PDK inhibitors in preventing increased blood glucose, reducing injury caused by myocardial ischemia, and inducing apoptosis of tumor cells. Regulations of PDKs expression or activity represent a very promising approach for treatment of metabolic diseases including diabetes, heart failure, and cancer. The future research and development could be more focused on the biochemical understanding of the diseases, which would help understand the cellular energy metabolism and its regulation by pharmacological effectors of PDKs.

## Introduction

**Figure 1:**
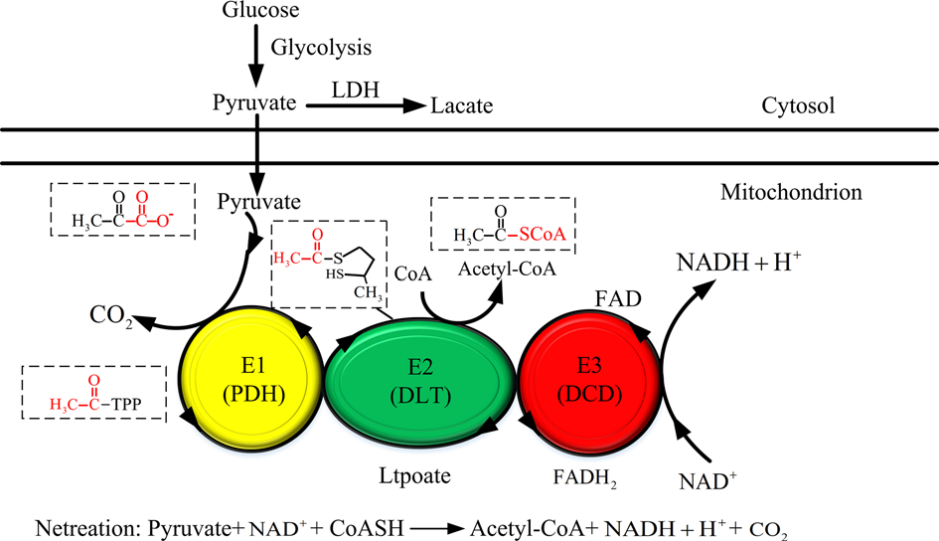
The mechanism of the pyruvate dehydrogenase complex Glucose is metabolized into pyruvate in the cytoplasm. Pyruvate passes through the inner mitochondrial membrane via the channel and carrier system. In mitochondria, pyruvate is irreversibly decarboxylated by the E1a subunit of heterotetrameric pyruvate dehydrogenase. E2 (dihydrolipidyl acetylase, DLT) transfers the acetyl group to the lipoic acid moiety, which can reduce coenzyme A (CoA) with acetyl. In the coupled redox reaction, fatty acid is reoxidized by E3 (dihydrolipidamide dehydrogenase, DLD) to generate NADH.

The pyruvate dehydrogenase complex (PDC) is the key enzyme system in the body that catalyzes the oxidative decarboxylation of pyruvate to form acetyl coenzyme A. By serving as a crossroad between glycolysis and the tricarboxylic acid cycle, PDC plays a crucial role in aerobic metabolism [1]. PDC is comprised by three catalytic enzymes and their regulatory proteins: pyruvate dehydrogenase (E1), dihydrolipoamide acetyltransferase (E2), and dihydrolipoamide dehydrogenase (E3) [2]. PDC in eukaryotic cells of higher animals (such as humans) has dihydrolipoamide dehydrogenase binding protein (E3BP) and two regulatory enzymes, pyruvate dehydrogenase kinase (PDK) and pyruvate dehydrogenase phosphatase (PDP), as shown in Figure 1. The two types of enzymes, PDK and PDP, strictly regulate PDC activity by phosphorylation (inhibition) and dephosphorylation (activation) of serine residues 293, 300, and 232 of the E1α subunit of heterotetramer pyruvate dehydrogenase [3]. PDKs, as the regulatory enzymes of pyruvate dehydrogenase (PDH), are located in the mitochondria, mainly distributed in mammals and play an important role in glycolysis.

In mammals, PDK1-PDK4 are expressed in tissue- or cell-type-specific ways by four different genes, which are responsible for tissue- or cell-specific regulation of PDC. They are mainly found in the mitochondrial matrix, and their sequence homology can reach to 70%. The sequence differences manifest mainly in the n-terminal. PDK1 is mainly distributed in the heart, islets, and skeletal muscles. PDK2 is widely expressed in many tissues, except for the spleen and lung. PDK3-expressing tissues limits to testicle, kidney, and brain. PDK4 is highly expressed in the heart, skeletal muscle, liver, kidney, and islets [4]. *In vitro*, various metabolites act as substrates for pyruvate dehydrogenase to regulate kinase activity. The tissue- or cell-specific expression of the PDK isoenzyme, as well as their specific activity and sensitivity toward effectors and metabolites, ensure synergistic regulation of glucose metabolism and hence the dynamic balance of glucose in the organism.

Under normal conditions, pyruvate dehydrogenase phosphatase inhibits PDK and activates PDC, which then catalyzes pyruvate in the tricarboxylic acid cycle to produce a large amount of ATP to meet the energy needs of the body. Pyruvate is a precursor of glucose synthesis and maintains blood glucose levels during starvation. When the body is under starvation, PDK inhibits the activity of PDC, leading to the failure of decarboxylation and accumulation of pyruvate in the cytoplasm. Under certain pathological conditions, phosphorylation of PDC inhibits the activity of PDK. Phosphorylation of PDK inactivates PDC, and pyruvate cannot be completely oxidized or converted into fatty acids. The malignant transformation of cells and changes in metabolic pathways continuously activate PDK. Enhancing the activity of PDC by inhibiting PDK provide potential drug targets for the treatment of diabetes, heart disease, and tumors. This article introduces the research status of the kinase activity regulation and related inhibitors, which will help understand its clinical application value.

## Regulation of pyruvate dehydrogenase kinase activity

### Phosphorylation and glucose metabolites

An important metabolic regulation mechanism of higher organisms is phosphorylation and dephosphorylation of enzymes. PDK is a Ser/Thr kinase, which phosphorylates the α subunit of PDH to inactivate PDH, which in turn inactivates PDC. Dephosphorylation of PDH by PDP can restore PDC activity [5]. Pyruvate dehydrogenase acts as a substrate for glycolysis and lactic acid cycle to produce pyruvate. Pyruvate, NAD+, and coenzyme A produced by glucose sugar metabolism inhibit PDK activity. The NADH and acetyl-CoA produced by the oxidative metabolism of glucose in mitochondria can activate the activity of PDK.

### Nutritional conditions and hormones

In starvation and diabetes, nutritional factors and hormones regulate the expression of PDK2 and PDK4, and the body reduces the activity of PDC by increasing PDK activity. Nutritional conditions and hormones can significantly change the expression of PDK and thus affect the physiological balance of glucose to fat.

Insulin [5], glucocorticoid [5,6], fatty acids [7], retinoic acid [8], and prolactin (through STAT5) [9] affect the expression of PDK, thereby regulating PDC, and controlling glucose and lipid metabolism. In untransformed mammary epithelial cells, matrix detachment markedly up-regulated PDK4 through estrogen-related receptor gamma (ERRγ), thereby inhibiting PDH and attenuating the flux of glycolytic carbon into mitochondrial oxidation. Also, depletion of PDK4 or activation of PDH increased mitochondrial respiration and oxidative stress in suspended cells, resulting in heightened anoikis. Conversely, overexpression of PDKs prolonged survival of cells in suspension [10]. ERRα could stimulate PDK4 expression, while one of the ERRα binding sites on PDK4 contributes to the insulin inhibition of PDK4. FoxO1-binding site on PDK4 is adjacent to the ERRα-binding sites, and FoxO1 participates in the glucocorticoid response of PDK4 and the regulation of PDK4 by insulin [5]. Glucocorticoids can effectively regulate blood sugar and increase the transcription of PDK4 gene, but insulin partially inhibits the induction of glucocorticoids by causing the dissociation of the glucocorticoid receptor and the promoter. Glucocorticoids bind to the FoXO1 promoter site through the glucocorticoid response element located at 820 bp upstream of the transcription start site to activate PDK4 expression. Ursula et al. [9] found that PRL and porcine GH can induce PDK4 expression in MC3T3-L1 adipocytes treated with prolactin (PRL), growth hormone (GH) or insulin, and insulin pretreatment can attenuate the induction ability of these hormones on PDK4 mRNA expression.

### Receptors affect PDKs expression

ERRs are orphan nuclear receptors that are involved in the transcriptional regulation of cellular metabolic pathways [11]. ERRα and ERRγ are expressed in metabolically active tissues such as skeletal muscle and liver. In liver, skeletal muscle and heart tissues, ERR stimulates PDK4 expression. The ERR-binding site is involved in the inhibitory effect of PDK4 on insulin. Peroxisome proliferation activated receptor mediates fibrin-induced PDK4 expression. In C2C12 cells, ERRα is involved in transcriptional activation of the PDK4 gene [12]. The transcriptional regulation of PDK4 by ERRα and ERRγ was also reported in the liver, which was associated with the recruitment of the peroxisome proliferator-activated receptor γ coactivator (PCG-1α) to the PDK4 promoter [11]. The Farnesoid X Receptor (FXR; NR 1H4) is a bile acids sensor and member of the nuclear receptor superfamily. FXR is expressed in neonatal cardiomyocytes and the treatment with FXR agonists increased the mRNA expression of FXR and its canonical target gene, as well as PPARα, acyl-CoA oxidase (AOX), and PDK-4 [13].

PPARs are a family of nuclear hormone receptors that function as transcription factors to regulate the expression of genes involved in metabolic pathways. PPARα, one of the main isoforms, is an important adaptive regulator during prolonged fasting that promotes ketogenesis and fatty acid oxidation, which is associated with increased expression of PDKs. In mice, treating with FXR agonists leads to an increase in PPAR expression, which in turn increases the expression of the mammalian tribbles homolog TRB-3. TRB-3 binds to Akt and inhibits the ability to phosphorylate targets (such as the transcription factor FoxO1), leading to increased expression of PDK4. Using PPARα-deficient mice with full, starvation and reflex, Mary C. Sugden et al. found that PPARα plays a key role in the adaptation of the kidney to fasting, and PDK4 is a downstream target of PPARα in the activation [14]. WY-14643, a special PPARα ligand, is a useful tool to identify PPARα regulatory genes [15,16]. Feeding PPARα null mice with WY-14643 activator leads to an increased expression of PDK4, indicating that PPARα activation is related to the regulation of PDK4 expression [17]. The expression of PDK4 in the tissues of starvation-treated wild-type mice was increased [18], but not in the tissues of PPARα null mice.

### Transcription factors affect PDKS expression

**Figure 2:**
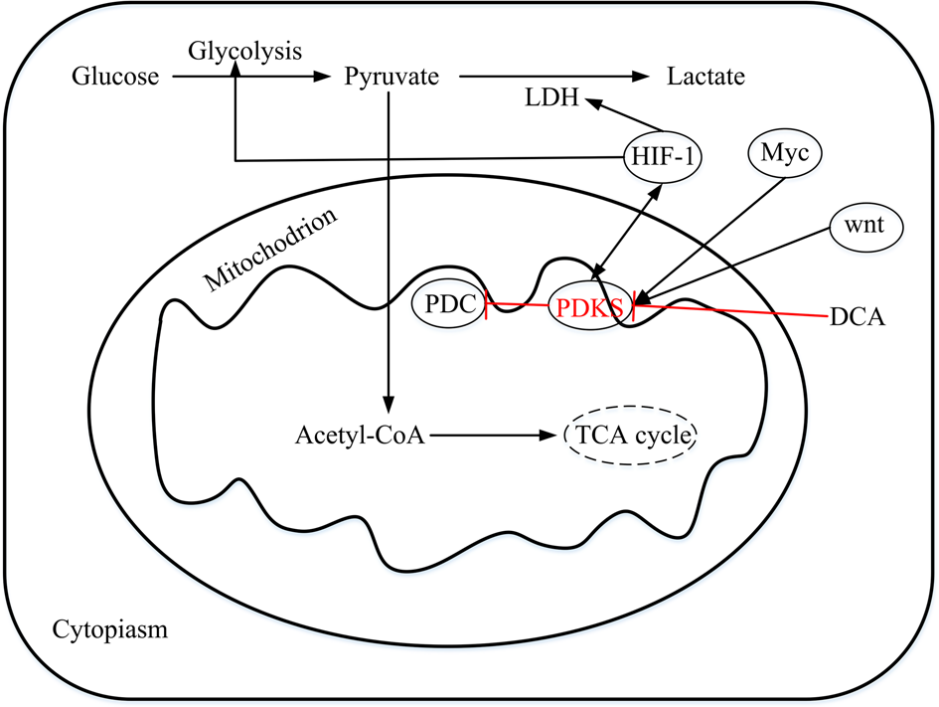
The potential roles of PDKs in malignant tumors The metabolic form of glucose in cells is pyruvate. Most of the pyruvate in noncancer cells enters the mitochondria under aerobic conditions, and a small part is metabolized into lactic acid. PDH in the mitochondria converts pyruvate into acetyl-CoA, which enters the TCA cycle. In tumor cells, the oxidative (mitochondrial) pathway for glucose utilization is inhibited, and most of pyruvate is converted to lactic acid. PDK inhibited by DCA inhibits PDH phosphorylation and regulates its activity. Myc, Wnt, and hypoxia-inducible factors (HIFs) individually or cooperatively transcribe one or more pyruvate dehydrogenase kinases in cancer cells. HIF-1 induces PDK to inactivate PDH and inhibits TCA circulation and mitochondrial respiration. HIF-1 can also stimulate the expression of glycolysis and LDH, thereby promoting the conversion of pyruvate to lactic acid.

Transcription factor (FoxO1) [19], hepatic nuclear factor 4 [20–22], Wnt [23,24], Myc [25,26], and hypoxia inducible factor (HIF) [26] are reported to have an impact on the expression of PDKs. In hypoxia and cancer, HIF1α activates the expression of PDK1 and PDK3. Under low-oxygen state, normal cells respond to a series of reactions, releasing hypoxia-induced factor HIF, which induces PDK expression and inhibits PDH activity. Mutants that directly or indirectly activating HIF signals are prevalent in cancer cells, and HIF further induces expression of PDK, resulting in a high expression of PDK in cancer cells [27,28]. Under hypoxia conditions, a variety of cellular signaling pathways controlling metabolism and survival are activated, and these signaling pathways also affect transcriptional expression of PDK4 as shown in Figure 2. Lee et al. [29] found that the co-expression of HIF-1α and HIF-1 β increased ERRγ promoter activity and mRNA expression under hypoxia conditions, thereby increasing PDK4 promoter activity and mRNA levels in HepG2 cells. ERR overexpression or knockout can significantly increase or reduce the expression of the PDK4 gene mediated by hypoxia.

FoxO1 [30] is a type of tumor suppressor transcription factor. As a transcription factor, nuclear FoxO1 regulate the expression of cell cycle and apoptosis-related genes. PDK1 is directly regulated by the proto-oncogene Myc. When MYC in malignant cells is amplified or abnormally activated, the expression level of PDK1 increases [25]. Wende AR et al. [12] found that PGC-1α, a key regulator of energy metabolism, activated the expression of ERRα in C2C12 skeletal muscle cells and combined with the PDK4 promoter to up-regulate of PDK4 mRNA and protein expression.

In summary, the activity of pyruvate dehydrogenase kinase is affected by many factors. The mutual restriction between nutrition and hormones, receptors and transcription factors can also promote the expression of PDK, thereby regulating the body’s physiological balance and metabolism.

## Application of pyruvate dehydrogenase kinase in clinical diseases

### Diabetes

PDC regulation in diabetes is important for glucose balance in mammals during feeding and fasting. Suppression of PDK triggers hunger [30]. In diabetic animals, PDC activity was inhibited by overexpressed PDK under starvation. This could limit excessive glucose consumption, leading to increased levels of blood glucose and protein glycosylation, ultimately causing damage to the cardiovascular system [31–34]. In obese individuals, insulin resistance caused decreased PDC activity, which is related to decreased glucose oxidation.

Abnormal regulation of PDC in diabetes is associated with two isoforms: PDK2 and PDK4. To inhibit high expression of PDK2 in the liver, Novartis and AstraZeneca [35] developed a new class of PDK inhibitors (Nov3r and AZD7545) against PDK2. These two inhibitors can increase and selectively activate glucose in muscle tissue. PDK inhibitors also regulate PDK4 activity to prevent the body from losing blood glucose during long-term starvation, and they also regulate PDC activity in glucose utilization and lipid metabolism.

Diabetes, fasting, and other conditions associated with the switch from the utilization of glucose to fatty acids as an energy source can up-regulate the expression of PDK4. Remarkable up-regulation of PDK4 has been reported in humans with Type 2 diabetes [36,37], in spontaneously diabetic Otsuka Long-Evans Tokushima Fatty (OLETF) rat diabetes [38], in animals and humans fed a high fat diet [39,40]. Peroxisome proliferator-activated receptor γ coactivator 1α (PGC-1α), one of the master regulator of energy metabolism, coactivates PDK4 gene expression through ERRα [12]. Using a high-fat (HF) diet induced insulin resistance in C57BL/6 J mice, Tuikka et al. [41] found that long-term high fatty acid intake activated PGC-1α and female ERRα, increased PDK4 expression, inhibited the effect of pyruvate dehydrogenase, and led to insulin resistance, suggesting that PDK4 is a possible contributor to high-fat diet-induced insulin resistance. In PDK4-deficient mice fed with high-fat diet, the expression of PDK4 in liver, kidney, skeletal muscle, and diaphragm was significantly reduced, fasting blood glucose levels were reduced, and glucose tolerance and insulin sensitivity were improved [42]. In contrast, in wild-type insulin resistant mice, high-fat feeding without PDK4 resulted in lower blood glucose levels and better glucose tolerance [43]. In the mouse model of Type 2 diabetes, liver insulin receptor substrates 1 and 2 are deleted, and additional knockout of PDK4 gene can improve blood glucose control and glucose tolerance [44]. PDK2/PDK4 double gene knockout mice cannot tolerate long-term fasting (48 h). These mice suffer from hypoglycemia, ketoacidosis, and hypothermia. These findings indicate that the activation of PDC can inhibit the activity of PDK, thereby reducing some symptoms of Type 2 diabetes. However, the complete activation of PDC by inhibiting phosphorylation is harmful and can even cause hypoglycemia and hypothermia to death [45,46]. Therefore, rational use of drugs that can affect PDK expression may have important application value in the treatment of diabetes

### Myocardial reperfusion

Myocardial damage caused by ischemia and reperfusion injury results in an imbalance between the production of oxidants and the availability of endogenous antioxidants [47]. Oxygen deprivation and ischemia leads to impaired mitochondrial function, increased mitochondrial division, heart damage, and eventually death. Reperfusion after an ischemic event can lead to another form of injury, that is, increased mitochondrial dysfunction and cell poptosis, necrosis, and heart failure. The functional status of PDK4 can change the myocardium’s choice of its metabolic substrates, which in turn affects myocardial energy metabolism. By inhibiting PDK4 activity to affect the stimulation of glucose oxidation, myocardial ischemia–reperfusion can reduce glucose oxidation, increase glucose uptake, and reduce myocardial ischemia–reperfusion injury.

Using an Angiotensin II (ANG II)-induced rat hypertrophy model, Mori et al. [48] found ANG II-treated hearts had a lower response to insulin with significantly reduced rates of glucose oxidation in association with increased PDK4 levels. Deletion of PDK4 prevented ANG II-induced diastolic dysfunction and normalized glucose oxidation to basal levels. The results suggest that inhibition of PDK4 (possibly activating SIRT3) can avoid myocardial insulin resistance caused by ANG, and may provide new treatment strategies for diastolic dysfunction and heart disease. Li et al. [49] established myocardial ischemia–reperfusion and hypoxia-reoxygenation models. PDK4 overexpression reduced glucose uptake, while PDK4 knockdown increased glucose uptake, indicating that PDK4 played a role in regulating glucose uptake. In addition, enhancement of glucose oxidation via inhibiting PDK4 contributes to the increase in glucose uptake. The results indicate that PDK4 stimulation of glucose oxidation may be an effective method to improve the recovery of myocardial ischemia–reperfusion injury. Olaniyi et al. [50] found that PDK4 plays a role in ethinylestradiol-levonorgestrel (EEL) formulation and/or high fructose-induced cardiac triglyceride (TG) and glycogen accumulation. Increased plasma and cardiac PDK4 was found in EEL or high fructose treated female Wistar rats while EEL or high fructose-induced alterations were ameliorated in EEL plus high fructose rats. The results demonstrate that high fructose-induced myocardial TG and glycogen accumulation is attributable to increased PDK-4. Therefore, EEL could be a useful pharmacological utility for protection against cardiac dysmetabolism by inhibiting PDK-4.

In recent years, increasing evidence has accumulated that the activity of PDH affects the recovery of myocardium after transient ischemia [51]. Tland et al. perfused isovolumically beating isolated rat hearts with erythrocyte-enriched buffer containing glucose, palmitate, and insulin, to investigate the effect of increasing degrees of ischemic injury on myocardial glucose oxidation and PDH activity during reperfusion [52]. PDH is activated after transient ischemia, whereby its activation is associated with the severity of the ischemic insult. In advanced ischemic injury, pronounced activation of PDH is also related to mitochondrial calcium uptake. In Type 2 diabetic patients with ischemia–reperfusion, elevated PDK4 activity may lead to insulin resistance in the body, and the improvement of myocardial reperfusion may stimulate glucose oxidation. Therefore, reducing myocardial injury may be achieved by increasing glucose uptake via inhibiting PDK4.

Adropin is a conservative peptide related to metabolic homeostasis and cardiovascular function [53–55]. Energy-expressing homeostasis-related genes (Enho) are highly expressed in the central nervous system, and they are also widely expressed in surrounding tissues such as liver, heart, skeletal muscle, and endothelium [56–58]. As a membrane-bound protein, adropin interacts with the notch signaling pathway to regulate cell-to-cell communication, and is involved in maintaining energy homeostasis and insulin response [59]. A comparison of whole-body substrate preference and skeletal muscle substrate oxidation in adropin knockout and transgenic mice showed that adropin promoted carbohydrate oxidation over fat oxidation. In these mice muscle, adropin activated PDH via down-regulating PDK4 and suppressed carnitine palmitoyltransferase-1B (CPT-1B), a key enzyme in fatty acid oxidation [54,55]. Adropin administration (*in vivo*) and direct addition of adropin to the perfusate of ex vivo hearts both induced a reduction in the inhibitory PDH and the protein levels of the responsible kinase PDK4. The pharmacological effects of adropin on energy metabolism and PDK4 in the heart and cardiomyocytes, indicating that adropin may be a putative candidate for the treatment of cardiac disease associated with impaired insulin sensitivity [56,60].

### Vascular calcification

Vascular calcification is caused by excessive deposition of calcium on the blood vessel wall. PDK4 plays a key role in vascular calcification and pyruvate oxidation, and helps maintain glucose balance in the body. Many factors that induce vascular calcification can also increase PDK4 expression, including autophagy [61], insulin [62], cholesterol changes [63], lipid metabolism [64], Runt related factors, and active oxidative stress [65–67].

Advanced glycation end products (AGEs) are derived from nonenzymatic reactions between sugars and the amino groups of protein, and are responsible for serious diabetic complications. Zhu et al. [68] found that AGEs are responsible for vascular smooth muscle cell (VSMC) calcification, which are the main cell type of vascular media. AGEs exposure could elevate HIF-1α and PDK4 expression levels in a dose-dependent manner, while PDK4 inhibition attenuated AGEs-induced VSMC calcification. Using a rat VSMC model, Ma et al. [69] proved that N ε-carboxymethyl lysine (CML), a major immunogen of AGEs, accelerated calcium deposition in VSMCs, while inhibition of PDK4 expression attenuated CML-induced VSMC calcification. Similar results were also reported by Sun et al. [70]. They found that PDK4 and pyruvate dehydrogenase complex phosphorylation was increased in calcifying vascular smooth muscle cells (VSMCs) and in calcified vessels of patients with atherosclerosis. PDK4 augmented the osteogenic differentiation of VSMCs by phosphorylating SMAD1/5/8 via direct interaction, which enhanced BMP2 signaling. These findings suggest that inhibiting PDK4 or impairing its function may be a new treatment strategy for vascular calcification.

Effective treatment of vascular calcification remains elusive. Dlamini et al. [71] found that inhibiting PDK4 can improve the vascular calcification of phosphate-treated vascular smooth muscle cells, aorta and vitamin D3-treated mice. Adenovirus released PDK4 (Ad-PDK4) overexpression in human vascular smooth muscle cells can significantly increase calcification and produce dependence. PDK4 can cause vascular calcification through mechanisms such as oxidative stress and apoptosis, and inhibiting PDK4 expression may be a putative strategy to prevent vascular calcification.

### PDKs and cancer

In different malignant tumors, the expression level and location of PDK isoenzymes are different, as shown in Table 1.

**Table 1:** Pyruvate dehydrogenase kinases and related pathological conditions

Subtypes of PDK	Related symptoms	Reference
PDK1	Human gallbladder cancer	[74]
	Breast cancer	[113]
	Ovarian cancer	[114]
	Hypoxic tumors	[115]
	Neck squamous cell carcinoma	[75]
	Multiple myeloma	[116]
	Colorectal cancer	[117]
	Glioblastoma	[118]
PDK2	Ovarian cancer	[119]
	Glioblastoma	[120]
	Type 2 diabetes	[121]
	Lung cancer	[84]
PDK3	Colon cancer	[87,88]
	X-linked Charcot-Marie-Tooth neuropathy	[122]
	Lung cancer	[123]
PDK4	Type 2 diabetes	[121,124,125]
	Hemochromatosis	[126]
	Glucocorticoid excess; e.g., Cushing syndrome	[5,127]
	Cardiac hypertrophy	[128]
	Statin induced myopathy	[129]
	Ovarian cancer	[119]
	Anoikis and tumor metastasis	[10]
	Vascular calcification	[68,69,130,131]
	Colon cancer	[91,132]
	Nonalcoholic steatohepatitis	[133]
	Prostate cancer	[134]

PDK1 is involved in the physiological regulation of transcription regulation, protein synthesis, cell migration, cell growth, differentiation, proliferation and apoptosis [72], and may be a viable cancer biomarker [73]. By increasing and decreasing PDK1 expression via plasmid transfection and siRNA administration in human gallbladder cancer (GBC) cell lines, Lian et al. [74] showed that PDK1 promoted the proliferation, invasion and migration of GBC cells by up-regulating JunB and epithelial–mesenchymal transition. In hypopharyngeal squamous cell carcinoma (HSCC), PDK1 is overexpressed, which is positively correlated with lymph node metastasis, clinical stage, and distant metastasis and indicated poor outcome. *In vitro* and *in vivo* study showed that PDK1 increased cell proliferation, migration, and invasion as well as tumor growth and metastasis. Hsu et al. [75] showed that in head and neck squamous cell carcinoma (HNSCC), EGF induced PDK1 expression. PDK1 knockdown repressed EGF-induced tumor cell transformation, and the downregulation of PDK1 expression or inhibition of its activity significantly blocked EGF-enhanced cell migration and invasion. Depletion of PDK1 impeded EGF-enhanced binding of HNSCC cells to endothelial cells as well as the metastatic seeding of tumor cells in lungs. These findings suggest that inhibition of PDK1 may be a potential strategy for the treatment of EGFR-mediated HNSCC metastasis. Gan et al. [76] found that nuclear localized PDK1 promoted breast cancer cell growth, migration, and invasion. Zhou [77] reported that in kidney cancer cells, expression of PDK1 was significantly up-regulated and overexpression of miR-375 in A-498 cells inhibited PDK1 via preventing the phosphorylation of AKT. Inhibition of PDK1 had similar effects as that of miR-375 overexpression on proliferation of A-498 kidney cancer cells. In colorectal cancer cells, PDK1 and PDK3 act as direct targets of histone lysine demethylase KDM4A and transcription factor E2F1 to regulate the glycolytic metabolism and mitochondrial oxidation [78]. Increasing evidence shows that Wnt signaling directs metabolic reprogramming of cancer cells to favor aerobic glycolysis or Warburg metabolism. In colon cancer cells, interference with Wnt signaling reduces glycolytic metabolism and results in small, poorly perfused tumors. PDK1, as an important direct target for metabolism, inhibits pyruvate flux to mitochondrial respiration. A rescue of PDK1 expression in Wnt-inhibited cancer cells rescues glycolysis as well as vessel growth in the tumor microenvironment [79,80]. High expression of PDK1 in both clinical samples and cell lines of ovarian cancer has been reported [81,82]. PDK1 expression was significantly associated with metastasis, reduced chemosensitivity, and poor overall and disease-free survival. Silencing of PDK1 retarded lactate production, cell adhesion, migration, invasion, and angiogenesis, and consequently metastasis. While overexpression of PDK1 showed converse effects, mainly through activation of JNK/IL-8 signaling. These findings support the efficacy of PDK1 as a valuable prognostic marker and therapeutic molecular target for cancers.

PDK2 is a key regulator of glycolysis and oxidative phosphorylation, and its expression is increased in a variety of tumors. The metabolism of glutamine and glucose is recognized as a promising therapeutic target for the treatment of cancer. In HepG2 and Hep3B cells, restricting the supply of glutamine markedly increased the expression of retinoic acid-related orphan receptor alpha (RORα). Overexpression of RORα or treatment with SR1078, the RORα activator, reduced aerobic glycolysis, down-regulated biosynthetic pathways, reduced PDK2 expression, inhibited the phosphorylation of pyruvate dehydrogenase, and subsequently shifted pyruvate to complete oxidation in hepatoma cells. Suppression of PDK2 inhibited hepatoma growth in a xenograft model [83]. In gastric cancer cells, miR-422a-PDK2 axis is an important mediator in metabolic reprogramming, providing a promising therapeutic target for antitumor treatment. MiR-422a repressed PDK2 to restore PDH activity, the gatekeeping enzyme that catalyzes the decarboxylation of pyruvate to produce acetyl-CoA. Mir-422a-PDK2 axis also influenced *de novo* lipogenesis in gastric cancer cells, which subsequently affected reactive oxygen species (ROS) and retinoblastoma protein (RB) phosphorylation levels, and cell cycle arrest. Increased expression of PDK2 was also found in paclitaxel-resistant lung cancer cells as compared with their parental cells [84]. Down-regulation of PDK2 can increase the sensitivity of drug-resistant lung cancer cells to paclitaxel, and combining paclitaxel with dichloroacetate (DCA), the specific PDK2 inhibitor, has a synergistic inhibitory effect on the viability of paclitaxel-resistant lung cancer cells. These findings highlight the importance of PDK2 for potential therapeutic interventions in patients who have developed a resistance to paclitaxel.

Similar to PDK1, PDK3 also participate in the metabolic switch of cancer cells, and has recently been considered as a potential pharmacological target for varying types of cancers. In metastatic melanomas, HIF-1/PDK3 axis functions as a sensor for metabolic stress, regulating mitochondrial ROS level under normoxia. HIF-1/PDK3 bioenergetic pathway is validated to be a new target for therapeutic intervention in metastatic melanomas. Pharmacologic or genetic blockades of the HIF-1α pathway decreased glycolysis and promoted mitochondrial respiration via specific reduction in the expression of PDK3. Inhibiting PDK3 activity by DCA or siRNA-mediated attenuation could increase pyruvate dehydrogenase activity, oxidative phosphorylation, and mitochondrial ROS generation [85]. In chemoresistant gastric cancer (GC) cells and gastric cancer tissues, PDK3 is highly expressed. Over-expression of PDK3 promotes the proliferation of GC cells while genetic or chemical inhibition of PDK3 could revert chemo-resistance *in vitro* and *in vivo* via forming a positive feedback loop with HSF1 (Heat shock factor 1) to drive glycolysis [86]. MiR-497-5p, a tumor suppressive microRNA in GC, inhibits GC cell proliferation and growth via directly targeting and suppressing the expression of PDK3 [87]. In colon cancer, PDK3 is markedly increased compared to that in adjacent normal tissues and PDK3 levels are positively associated with severity of cancer and negatively associated with disease-free survival. In colon cancer cell lines, PDK3 expression is controlled by HIF-1α and contributes to hypoxia-induced increased drug resistance [88,89]. PDK3 plays an important role in the metabolic switch and drug resistance, and is potentially a novel target for cancer therapy.

PDK4 silencing can enhance cell proliferation and tumorigenesis. Qin et al. [90] showed that PDK4 silencing can increase the migration and invasion of BEL-7402 and BEL-7404 cells *in vitro*, and the loss of PDK4 expression was related to the malignant progression of liver cancer cells. Up-regulation of PDK4 enhances the resistance of hepatocytes and colon cancer cells to chemotherapy-induced toxicity, while down-regulation of PDK4 enhances chemotherapy-related cell damage. PDK4 is up-regulated in normal mucosa of patients with colorectal cancer (CRC), and down-regulation of PDK4 in human colon cancer cells reduces cell migration and invasion, and increase cell apoptosis [91]. Trinidad et al. [92] found that knockout of PDK4 could down-regulate the expression of the mutant oncogene KRAS, thereby inhibiting cell growth of lung cancer and colorectal cancer (CRC) cells, indicating that PDK4 is an attractive target for cancer treatment.

In summary, pyruvate dehydrogenase kinases, as key node kinases that control glycolysis and oxidative phosphorylation pathways, play an important role in tumor cell proliferation, invasion, migration, apoptosis resistance and metabolic switch, providing new therapeutic opportunities against different types of cancers.

## PDKs inhibitors

The high expression of PDK isoenzymes in tumor tissues is closely related to abnormal glycolysis in tumors, especially PDK1, which is the only kinase that can phosphorylate the three phosphorylation sites of E1 [93] and is related to the prognosis of cancer patients. It is closely related to survival, response to radiotherapy and chemotherapy, and development of drug resistance. The use of PDK inhibitors has been widely spread as a potential therapeutic in laboratory models of multiple cancers. Currently, efforts are still underway to generate more selectively PDK-specific inhibitors with smaller side effects, and to delineate the roles of individual PDK isozymes in specific cancers and other diseases [94].

At present, there are mainly four types of PDK inhibitors, which were classified according to different binding sites. The first category is inhibitors that act on the binding site of pyruvate, such as dichloroacetic acid (DCA). DCA is the only PDK inhibitor that has entered phase 2 clinical trials, but the side effects, such as weak anticancer activity and excessive drug dose (100 mg/kg) restrict its clinical application [95]. Mechanistically, DCA is a pyruvate analog that promotes conformational changes at the active-site cleft of PDK1 and hinders the dissociation of ADP from the active site [96,97]. A phase I clinical study of 15 adult gliomas with recurrence or primary cancer metastasis showed no dose-limiting toxicity of oral DCA. The genetic dose has been confirmed and included in the future chronic DCA management trial [98]. In a phase II clinical study of 10 multiple myeloma patients, parenteral nutrition was the only significant side effect after long-term use and was reversible. DCA at 6.25 mg/kg b.i.d did not seriously worsen parenteral nutrition, so DCA can be used for patients with peripheral neuropathy [99]. Despite the therapeutic and economic benefits of DCA, it has been used in limited situations, such as life-threating lactic acidosis induced by PDH-E1α deficiency or sepsis for the short term because of its toxicities, including neuropathy and hepatic tumorigenesis, poor pharmacokinetics, and low potency and selectivity [100].

The second category is inhibitors that act on dilipoic acid pocket compounds, such as Nov3r, AZD7545, and CP1613 [96,101–103]. AZD7545 is a drug developed by AstraZeneca to treat Type 2 diabetes [104]. It inhibits the activity of PDHK1-2 by inserting the lipoyl binding pocket of PDHK1 and PDHK2, and has no inhibitory effect on PDHK4. *In vivo*, AZD7545 can selectively inhibit PDHK2, activate PDH activity, and improve blood glucose levels [96,105,106]. It has been discontinued in clinical trials for reasons that have not been reported. CPI-613, a lipoate derivative, inhibits the entry of glucose and glutamine derived carbons through its inhibition of pyruvate dehydrogenase and a-ketoglutarate dehydrogenase complexes. It has been investigated in phase I clinical trials for patients with relapsed or refractory hematological malignancies [101]. In a Phase I trial for patients with previously untreated metastatic pancreatic cancer, CPI-613 has shown promising results when combined with modified FOLFIRINOX (mFFX) [107]. A phase III open-label trial to evaluate the efficacy and safety of CPI-613 plus modified FOLFIRINOX (mFFX) versus FOLFIRINOX (FFX) in patients with metastatic adenocarcinoma of the pancreas is under investigated [108]. However, in a phase II clinical trial of CPI-613 in patients with relapsed or refractory small cell lung carcinoma, single agent CPI-613 had no efficacy [109]. The clinical side effects of CP1613 also need to be studied.

The third category is an ATP-competitive inhibitor, such as radicicol and M77976 [96,110]. Radicicol can directly bind to the ATP-binding pocket of PDK to inhibit its activity. In one hand, the ATP pocket of PDK3 of radicicol is very similar to Hsp90 in structure, and the inhibitory activity for Hsp90 is very strong, at 20 nmol/l, while the effective concentration for inhibiting PDK is only μmol/l level. In the other hand, the biological activity *in vitro* is good, but its stability and antitumor activity *in vivo* is poor [96]. The high similarity of the ATP-binding pocket of kinases may lead to off-target effects, resulting in side effects and toxicity issues, which restrict their clinical use.

The fourth category is a covalent modifying inhibitor, which acts on the binding pocket near the PDK isoenzyme cysteine residue (C240) and modifies it covalently, such as JX06 [111]. By changing the conformation of PDKs, this kind of inhibitors reduces ATP affinity of PDKs, thereby effectively inhibiting the activity of PDK1. JX06 recognizes a hydrophobic pocket in PDK1 and covalently binds to a conserved cysteine residue of PDK1 [111]. This covalent modification impairs PDK1 enzymatic activity via reducing the ATP affinity of PDK1. These findings prove the possibility of the rationale design of PDK1 covalent inhibitors.

In summary, the current four types of PDK inhibitors all have deficiencies. Radicicol is inactive in the body, AZD7545 has been discontinued in clinical trials for unknown reasons, the activity of DCA is weak, and the effective concentration is millimolar, while JX06 faces the challenge of potency, selectivity, efficacy, and resistance. There are other drugs that have inhibition on PDKs. The anti-inflammatory drug aspirin inhibits the level of glycolysis by inhibiting the expression of PDK1 and reducing the glucose uptake, lactic acid production and ATP levels of tumor cells, thereby killing breast cancer stem cells [112]. These new and old inhibitors provide many new ideas and strategies aiming at targeting PDK for anticancer therapy and treatments for other metabolic diseases.

## Conclusions and prospects

Ongoing investigations of the central role of PDC and PDK in cellular energy metabolism and its regulation by pharmacological effectors open multiple exciting vistas into the biochemical understanding and treatment of cancer and other metabolic diseases. This study reviewed the regulation mechanisms and application of PDK in different species, and demonstrated the inhibitors of PDK regulation and PDK isoenzymes that can be used to treat Type 2 diabetes, myocardial ischemia, and other diseases caused by abnormal glucose metabolism.

Given the many pharmacological effects involved in PDK, it may be a potential target for antitumor drugs and metabolic disease treatments. Despite important therapeutic prospects, the development and application of PDK targeting drugs in clinical practice still face many challenges. The existing findings are mainly limited to animals and cells, and further clinical trials are needed. It is necessary to design and synthesize efficient and low-toxicity PDK inhibitors, which also presents considerable challenges for laboratory research and subsequent clinical trials.

## Abbreviations

CML: carboxymethyl lysine
CRC: colorectal cancer
DCA: dichloroacetate
DLD: dihydrolipoamide dehydrogenase
DLT: dihydrolipoyl transacetylase
EGF: epidermal growth factor
ERR: estrogen-related receptors
FoxO1: Forkhead transcription factor
GBC: gallbladder cancer cells
GR: glucocorticoid receptors
HIF: hypoxetic induction factors
LDH: lactate dehydrogenase
OXPHOS: oxidative phosphorylation
PDC: pyruvate dehydrogenase complex
PDH: pyruvate dehydrogenase
PDK: pyruvate dehydrogenase kinase
PDP: pyruvate dehydrogenase phosphatase
RORα: retinoic acid-related receptor α
TCA: tricarboxylic acid
VSMC: vascular smooth muscle cell
